# Mapping blood vessels and oxygenation throughout the whole mouse brain with dual-frequency fiber-array photoacoustic computed tomography

**DOI:** 10.1038/s41377-026-02440-0

**Published:** 2026-08-18

**Authors:** Gen Mu, Yanyu Zhao

**Affiliations:** Beijing Advanced Innovation Center for Biomedical Engineering, Key Laboratory for Biomechanics and Mechanobiology of Ministry of Education, School of Engineering Medicine, Beihang University, 100191 Beijing, China

**Keywords:** Imaging and sensing, Optical sensors

## Abstract

A new all‑optical photoacoustic tomography system captures both low‑ and high‑frequency photoacoustic signals using an arc‑shaped fiber array. The high‑frequency channel resolves cortical vessels as fine as 70 μm near the surface; the low‑frequency channel reaches 1.2 cm to image the entire mouse brain. With ultrahigh sensitivity and blood oxygen saturation mapping, this dual‑frequency design alleviates the classic trade‑off between resolution and penetration—offering high‑resolution surface imaging and deep, wide‑field coverage within a single imaging platform.

Accurately mapping the 3D structure and oxygenation of cerebral vessels is essential for diagnosing brain disorders^1^. Conventional imaging methods such as magnetic resonance imaging (MRI), computed tomography (CT), and ultrasound are limited by metal implants, radiation, and a lack of oxygenation information, respectively^2–4^. In contrast, photoacoustic computed tomography (PACT) offers a powerful alternative by combining optical contrast with ultrasound penetration to map vascular structure and oxygen saturation using endogenous hemoglobin contrast^5–7^. Yet existing PACT systems are constrained by narrow bandwidth, bulky rigid transducers, and fixed geometries^8–12^. For brain imaging, these limitations force a trade-off between fine cortical resolution and deep penetration. What is urgently needed is a highly sensitive PACT system that captures both high‑frequency signals for superficial microvessels and low‑frequency signals for whole-brain depth, with a flexible array geometry.

To meet the demands outlined above, the research team led by Bai-Ou Guan and Yi Zhang at Jinan University has developed a dual-frequency fiber-array photoacoustic computed tomography system^13^, as shown in Fig. 1. The system features an arc-shaped ultrasound detection array composed of eight fiber laser cavities. Each fiber laser cavity converts ultrasound strain into a beat frequency shift that is demodulated to recover the acoustic signal^14,15^, achieving a detection limit of 5.2 Pa, an imaging depth of 1.2 cm, and a cortical resolution of about 70 μm, capable of mapping blood oxygen saturation across the entire mouse brain.

**Fig. 1:**
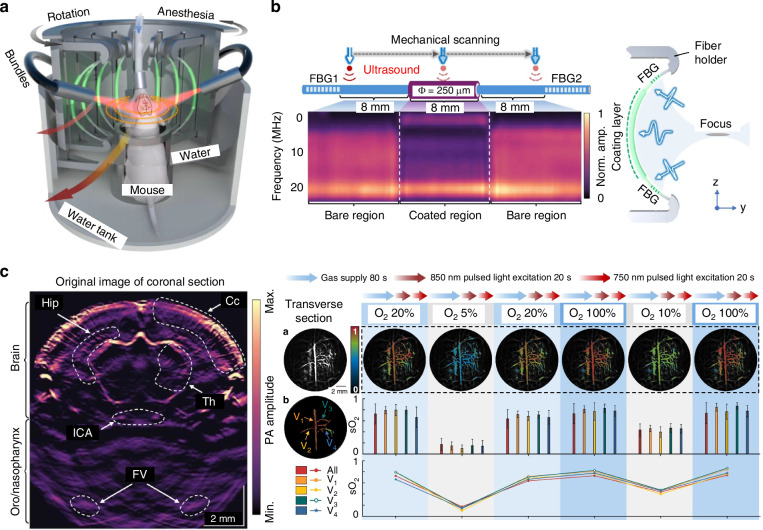
Dual‑frequency fiber‑array photoacoustic computed tomography (PACT) visualizes whole‑brain vasculature and oxygen saturation. **a** System schematic of the dual-frequency fiber-array PACT. **b** Frequency response map of a partially polymer-coated cavity between FBG1 and FBG2. The schematic on the right illustrates the mechanically bent fiber forming a sheet-like ultrasound focus. FBG fiber Bragg grating. **c** Left, original image of coronal section. Hip hippocampus, Cc cerebral cortex, ICA internal carotid artery, Th thalamus, FV facial vessel. Scale bar: 2 mm. Right, transverse section cerebral sO_2_ maps during sequential exposure to different O_2_ concentrations. The bar plots (upper) show the mean ± SD sO_2_ values for the entire segmented vascular region and for the four individual vessels indicated by the arrows, whereas the line plots (lower) show the corresponding mean sO_2_ values. Scale bar: 2 mm

At the heart of the system is a fiber‑optic ultrasound sensor that can be bent into a sheet‑like focus and captures both low and high frequencies. The dual-frequency response arises from the distinct frequency response spectra of bare and polymer-coated fiber: the bare fiber is sensitive to high-frequency signals, while the coated fiber responds to the low-frequency range. By adjusting the length ratio of the two fiber sections, the response curve can be tuned, and with filtering, dual-frequency detection in the regions near 2.5 MHz and 20 MHz is achieved. Bending the fiber creates a sheet-like focus with high sensitivity, while varying the curvature radius adjusts the field of view and response amplitude—though the two are trade-offs.

Coronal section imaging of the mouse brain showed that the dual‑frequency design delivers two complementary views. The high-frequency channel resolves cortical vessels down to about 70 μm near the surface; even at 4 mm depth, vessels of 130 μm are still resolvable. The low-frequency channel, by contrast, sees much deeper (up to 1.2 cm) covering the entire brain as well as the oronasopharyngeal region. Together, the two channels allow the system to penetrate the whole mouse brain and simultaneously resolve cross‑scale vascular networks across the cortex, hippocampus, and thalamus.

In addition to structural information, the system also delivers functional information. In vitro experiments on blood oxygen saturation revealed that high-frequency signals only capture localized oxygen saturation near the vessel wall interface, with considerable uncertainty. In contrast, low-frequency signals accurately measure the oxygenation level of the entire blood flow, and the results are consistent with those from a commercial blood gas analyzer. The team then monitored cerebral sO₂ dynamics in mice for breathing different oxygen concentrations. Throughout these experiments, the oxygenation trends in representative vessels closely followed the average trend across the entire imaging region, demonstrating the system’s ability to accurately map changes in cerebral blood oxygen saturation. When imaging subcutaneous tumors and orthotopic glioblastomas, the researchers observed complex, highly branched vessels and elevated oxygen saturation within the tumor regions. Those findings align well with MRI and histopathological results.

The dual-frequency fiber-array PACT system demonstrates remarkable capabilities in imaging both the structure and function of mouse cerebral vasculature. The fiber-optic transducer, with a diameter of less than 1 mm, achieves highly sensitive detection of weak acoustic signals. With its ability to simultaneously capture low- and high-frequency signals—with tunable frequency characteristics—the system enables cross-scale structural and functional imaging in a single setup. Moreover, because the fibers are flexible and can be bent and arranged into arbitrary array geometries, the technology holds promise for a wide range of applications, including whole-body imaging of small animals, human brain imaging, extremity and breast imaging^16–19^, as shown in Fig. 2. Additionally, due to its small size, flexibility, and portable nature, one should combine the presented transducer with carefully designed acoustic encoder and compressive sensing, so that one can potentially achieve snapshot photoacoustic tomography of the whole mouse brain for free-behaving mice^20–22^. That being said, the current system also has certain limitations. It relies on mechanical rotation of only eight fibers, giving a relatively slow acquisition speed. Increasing the number of array elements could potentially overcome this limitation and enable real-time imaging. Additionally, the imaging depth would be further improved by using shortwave infrared wavelengths^23–25^.

**Fig. 2:**
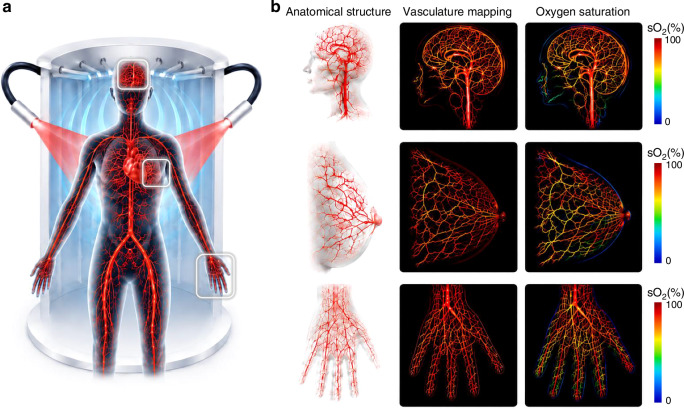
Application prospects for human brain, breast and extremity imaging. **a** Conceptual workflow for cross-scale whole-body imaging using a reconfigurable fiber-array PACT system. The white boxes indicate representative regions of interest in the brain, breast, and extremity. **b** Conceptual anatomical structures and corresponding prospective vascular and oxygen saturation maps for the human brain (top), breast (middle), and extremity (bottom)

Overall, the proposed PACT system delivers high resolution, large penetration depth, and versatile functional imaging—pointing toward promising clinical translation and potential cross-scale imaging of human organs.
